# NMR-Based Metabolomics in Differential Diagnosis of Chronic Kidney Disease (CKD) Subtypes

**DOI:** 10.3390/metabo12060490

**Published:** 2022-05-28

**Authors:** Styliani A. Chasapi, Evdokia Karagkouni, Dimitra Kalavrizioti, Sotirios Vamvakas, Aikaterini Zompra, Panteleimon G. Takis, Dimitrios S. Goumenos, Georgios A. Spyroulias

**Affiliations:** 1 Department of Pharmacy, University of Patras, 26504 Patras, Greece; stella.chimic@gmail.com (S.A.C.); evkaragkounh@gmail.com (E.K.); azompra@upatras.gr (A.Z.); 2 Department of Nephrology and Renal Transplantation, University Hospital of Patras, 26504 Patras, Greece; dkalavrizioti@yahoo.com (D.K.); sotvam74@gmail.com (S.V.); 3 Section of Bioanalytical Chemistry, Division of Systems Medicine, Department of Metabolism, Digestion and Reproduction, Imperial College London, South Kensington Campus, London SW7 2AZ, UK; p.takis@imperial.ac.uk; 4 National Phenome Centre, Department of Metabolism, Digestion and Reproduction, Imperial College London, Hammersmith Campus, IRDB Building, London W120NN, UK

**Keywords:** CKD, urine, metabolomics, NMR spectroscopy, glomerulonephritis, IgA, membranous, eGFR

## Abstract

Chronic Kidney Disease (CKD) is considered as a major public health problem as it can lead to end-stage kidney failure, which requires replacement therapy. A prompt and accurate diagnosis, along with the appropriate treatment, can delay CKD’s progression, significantly. Herein, we sought to determine whether CKD etiology can be reflected in urine metabolomics during its early stage. This is achieved through the analysis of the urine metabolic fingerprint from 108 CKD patients by means of Nuclear Magnetic Resonance (NMR) spectroscopy metabolomic analysis. We report the first NMR—metabolomics data regarding the three most common etiologies of CKD: Chronic Glomerulonephritis (IgA and Membranous Nephropathy), Diabetic Nephropathy (DN) and Hypertensive Nephrosclerosis (HN). Analysis aided a moderate glomerulonephritis clustering, providing characterization of the metabolic fluctuations between the CKD subtypes and control disease. The urine metabolome of IgA Nephropathy reveals a specific metabolism, reflecting its different etiology or origin and is useful for determining the origin of the disease. In contrast, urine metabolomes from DN and HN patients did not reveal any indicative metabolic pattern, which is consistent with their fused clinical phenotype. These findings may contribute to improving diagnostics and prognostic approaches for CKD, as well as improving our understanding of its pathology.

## 1. Introduction

Chronic Kidney Disease (CKD) describes a clinical entity, which is caused by many different pathogenies (glomerulonephritis, hypertensive nephrosclerosis, chronic interstitial nephritis, diabetic nephropathy, etc.) and is influenced by both genetic and environmental factors [1]. Individuals diagnosed with CKD are usually subjected to aggressive treatment strategies to attenuate the degree of disease progression, which can ultimately lead to end-stage kidney failure. Early diagnosis and treatment can significantly delay the progression of chronic kidney disease. Until now, the urinary albumin excretion, the value of estimated Glomerular Filtration Rate (eGFR), which is based on serum creatinine (less than 60mL/min/1.73m^2^ (for over three months)) and the blood urea nitrogen are the most widely used biomarkers used for the identification and classification of the normal function of the renal system [2,3]. However, none of them can predict or indicate the primary cause of kidney damage or be used as a marker of CKD progression. Conventionally, CKD cause is assigned based on the presence or absence of underlying systemic diseases and the location of known or inferred anatomic abnormalities. The etiology discrimination between a systemic and a primary (also called idiopathic) kidney disease is based on the origin and the exact anatomical position of the disease process [4].

Kidney diseases have been classified as having four categories: (i) glomerular diseases; (ii) tubulointerstitial diseases; (iii) vascular diseases; and (iv) cystic and congenital diseases [5]. Overall, the three etiologies chiefly associated with CKD are glomerulonephritis, hypertension and diabetes. In populations with a high prevalence of diabetes and hypertension, it is difficult to distinguish whether CKD was caused by one of these morbidities from the occurrence of CKD, or due to other pathologies. Most of the time, the presence of a specific etiology might also be driven by factors such as race and geographical location that are directly associated with well-being, diet and disease.

In this study we investigate the way that the three most prevalent etiologies (glomerulonephritis, diabetic nephropathy, and hypertensive nephrosclerosis) are depicted in urine metabolome either as a result of idiopathic disease or as a systemic disease. Regarding the first etiology, glomerulonephritis, we focus solely on the two major types of glomerulonephritis, which are diagnosed mainly through biopsy, Membranous (MN) and IgA Nephropathy (IgAN) [6,7]. Diagnosis of MN is performed mainly through renal biopsy following the initial laboratory and radiological interventions (imaging tests) [8]. The major disadvantage in this case is that renal damage is only verified once the disease’s onset and the lack of predictive biomarkers make the prevention and progression of the disease a difficult task. Herein we first report differential urine metabolome pattern regarding the etiology of CKD patients from the region of Western Greece. The purpose of this study is to explore the range of biochemical information that can be provided by urine fingerprinting and how may be applied in clinical practice and laboratory testing [9]. Urine metabolome is imprinted using the non-destructive analytical platform of a high-resolution NMR spectrometer and the last decade is gaining high reliability [10,11]. Throughout this study, urine metabolome is defined by the ^1^H NMR spectrum of a urine sample that was collected at a given time point per individual. Since the biochemical information regarding the urine’s composition in metabolites of small molecular weight (<1500 Da) and concentrations is time-dependent, we strongly believe that this analytical tool may provide significant clinical information about the pathophysiological status of an individual by monitoring its urine metabolic fingerprint between frequent time intervals. This is not the first time that NMR spectrometry is presented as an analytical tool for urine metabolomics, deciphering the metabolic alterations of CKD pathology and subtypes. Recently two studies target the two most common glomerulonephritis subtypes, providing potential classification models and biomarkers. The studies by Park, Sehoon, et al. [12] and Taherkhani, Amir, et al. [13] provide the first, comprehensive metabolomic studies of IgAN and primary MN, respectively, providing first insights into the underlying causes of each condition.

Hypertensive Nephrosclerosis (HN) and Diabetic Nephropathy (DN) are among the major causes of CKD and were selected in the present study [14,15]. DN occurs in patients with Type 1 and 2 diabetes mellitus several years after the onset of the primary disease. Both CKD subtypes are usually diagnosed by clinical findings such as eGFR decline and albuminuria, while biopsy testing is limited in performance [16,17,18,19]. Clinical routine has shown that using only these clinical values, HN and DN are indistinguishable. However, DN individuals present an annual GFR decline four times greater than that of HN. For this reason, etiology confirmation in CKD patients who have both diabetes and hypertension has become an obstacle for clinicians since they both may lead to end stage renal disease (ESRD). Hence, it is of great importance to reliably predict who is going to rapidly progress to ESRD [20].

So far, several targeted metabolomic analyses on different types of samples (urine, plasma, serum), have shown changes in the urine metabolic profiles of patients with CKD and other pathological conditions [21,22,23,24,25]. Identification of metabolites-markers or indicators of progression and stage evolution, through NMR metabolomic analysis of urine samples from CKD patients, will have great impact and may prospectively facilitate the diagnosis of the cause of CKD and timely treatment. NMR metabolomic analysis can provide information that can be used to pinpoint future classification strategies for chronic kidney disease populations.

## 2. Results

A total of 60 metabolites were successfully detected and assigned in urine NMR spectra from CKD patients (Table 1). Due to the heterogeneity of the CKD causes and the individual contribution (fitness, medications and diet) on the urine metabolome only the metabolites with ^1^H NMR discriminant signals were examined for statistical significance. Untargeted analysis of the urine metabolome was performed analyzing the entire 1D ^1^H NMR spectrum of each individual, accounting every single change in urine metabolites composition. Furthermore, classification models and multivariate statistical analysis of the binned NMR data was based on the reported chemical shifts to associate statistically important spectral regions with specific metabolites.

### 2.1. Glomerulonephritis. Membranous versus IgA Nephropathy

**Figure 1:**
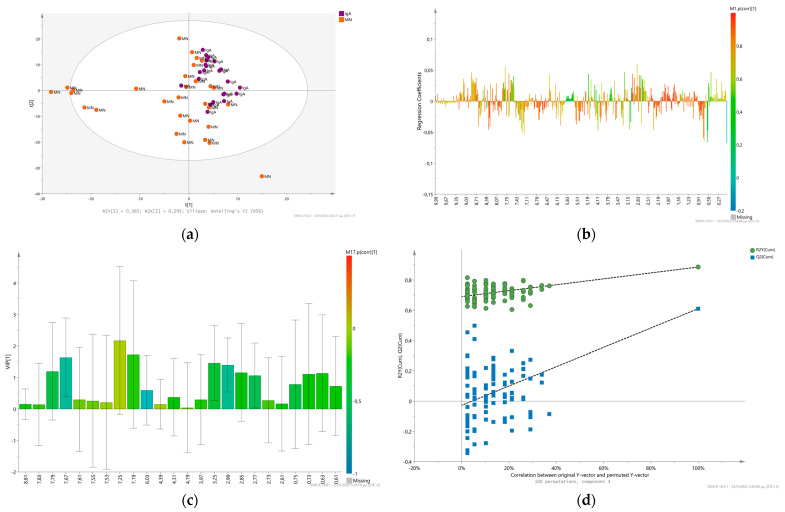
Multivariate analysis of ^1^H 1D NMR urine spectra of patients with the two highly occurring etiologies, IgAN (purple spheres) and MN (orange spheres). (**a**) PLS-DA scores plot of the IgAN and MN glomerulonephritis. (**b**) PLS-DA Coefficients’ plot of the comparison between the CPMG urine spectral data of IgAN and MN glomerulonephritis. (**c**) PLS-DA VIP scores first 25 in sort descending order of urine glomerulonephritis metabolomes colored according to their presenting correlation and contribution coefficients “p(corr)” at the first latent component VIP [1]. (**d**) Permutation plot of the PLS-DA model validating the statistical significance of the model as the permuted R^2^ (green cycles) and Q^2^ (blue squares) are lower than the original values of R^2^ and Q^2^.

To visualize differences between the urine metabolic profiles collected from CKD patients with different etiologies of Glomerulonephritis, PCA analysis and HCA were conducted as unsupervised multivariate methods. Examination of the urinary metabolic space from patients diagnosed with idiopathic MN show a variability regarding the metabolome of other glomerulonephritis patients. There is a wide spectrum of differences in the urine metabolic fingerprint of patients within the population. MN patients present high urinary metabolic heterogeneity irrespective of the type of treatment and eGFR value, compared to the other examined types of glomerulonephritis (IgAN and control diseased). In Figure 1a, the PCA-class model for MN patients present R^2^X(cum): 0.756 and Q^2^(cum): 0.649 on the third principal component. In the 2D PCA scores plot, spheres represent the ^1^H NMR urine fingerprint of patients diagnosed with MN and are colored due to their eGFR value (Figure S1a). The PCA-Hierarchical model is also performed to investigate the non-linear behavior of the data (Figure S1b). Hierarchical cluster analysis reveals four sample groups within MN glomerulonephritis using Ward’s method to calculate the proximity of the clusters, following a divisive approach and with trees sorted by size.

IgA Nephropathy patients present minor variability within their population group. The PCA class model (R^2^X(cum): 0.593, Q^2^(cum): 0.346) and HCA indicate two clusters within the IgAN population (Figure S2).

We attempted to differentiate MN patients from IgAN based on their urinary metabolic profile, using the supervised method PLS-DA. Initially, the OPLS-DA model was tested, using five orthogonal components in X, however evaluation by a seven-fold cross validation resulted in an over-fitted model (R^2^Y(cum): 0.94 and Q^2^(cum): 0.542, R^2^Y intercept: 0.821, Q^2^ intercept: −0.623). The PLS-DA model constructed using four LVs present a good classification with R^2^X(cum): 0.72, R^2^Y(cum): 0.885 and Q^2^: 0.61 (Figure 1a) and permutation plot (Figure 1d) resulted in R^2^Y intercept: 0.696, Q^2^ intercept: 0.00269. The first 23 higher VIP scores ranging from 2.1 to 1.55 (Figure 1c) indicate that Trigonelline (8.81), Hippurate and its intense chemical shift variation (7.67, 7.83, 7.79), Hippurate (7.55, 7.53), Indoxyl sulfate (7.25), *N*-phenylacetylphenylalanine (7.19), NAD^+^ (6.03), uridine (4.39), Tartate (4.31), pyroglutamate (4.19), Glycolate and Hippurate (3.97), TMAO (3.25), lysine and gamma-aminobutyrate (2.99), aspartate and overlapping ^1^H peaks (2.85), NADH/NADPH (2.77), citrate (2.73, 2.61). Additionally, ^1^H NMR signals of Lisinopril (drug administration of Zestril) was found to contribute highly in the ^1^H NMR spectra (multiplets at 7.40, 7.33 and 2.33 ppm) of the patients who received this medication (Figure 1b).

To evaluate further the importance of metabolites detected in discriminating the two metabolomes, IgAN and MN, ROC analysis was applied to the NMR data. Predicted importance of the PLS-DA model showed that areas under the curve (AUC) were 0.866 for MN patients and 0.965 for IgAN.

#### Univariate Analysis-Biomarker Evaluation on IgAN and MN Discrimination

**Figure 2:**
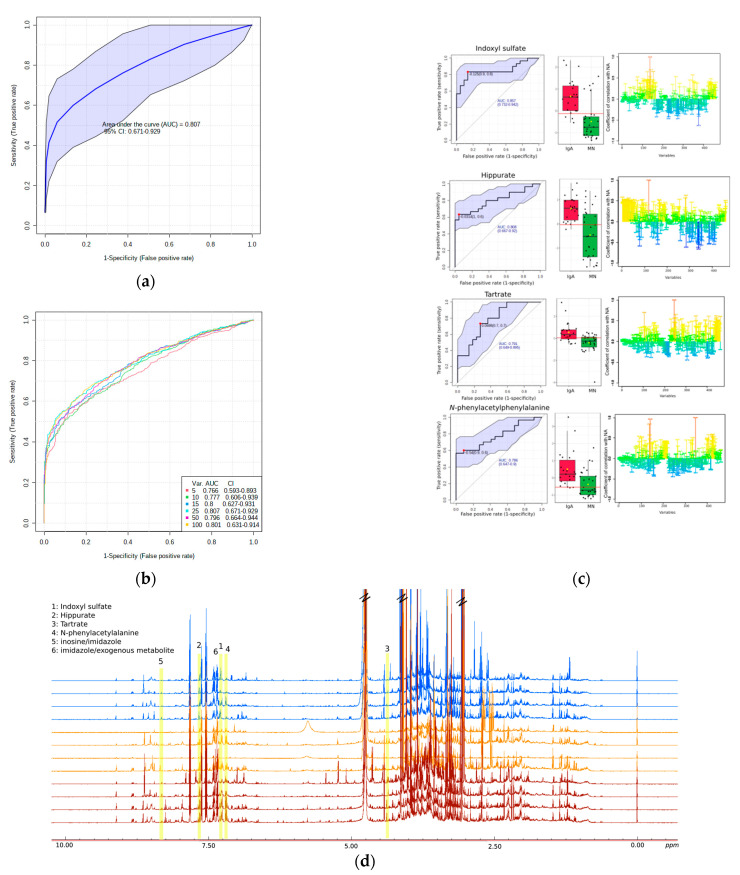
(**a**) The ROC curve computed based on all the 25 features with the 95% confidence interval; (**b**) Multivariate receiver operating characteristic (ROC) analysis, showing the feature numbers the AUCs and the confidence intervals of the six models and; (**c**) The top four biomarker candidate metabolites identified based on ROC curve analysis performed with all 25 urine metabolic features. The computed 95% confidence interval (CI) for individual marker metabolites is highlighted in the faint blue background over the ROC curve. The area under the receiver operating characteristic curve (AUROC) is shown in red to highlight the diagnostic potential of corresponding metabolite. The box-cum-whisker plots shown on the right side of each ROC curve plot revealed significantly increased urine levels of these metabolites in the IgAN patients compared to MN. For each metabolite the representative 1D STOCSY pseudo-NMR spectrum of correlation coefficients to the other signals in the median urine NMR spectrum and maximum intensity correlation of peaks are color encoded and projected into statistical difference spectra; (**d**) Representative ^1^H CPMG NMR spectra of urine samples of MN (blue spectra), IgAN (brown spectra) patients and healthy controls (orange spectra) and the corresponding metabolites of interest (^1^H NMR signals) highlighted in yellow.

To evaluate the identified metabolites regarding their diagnostic accuracy, they were further subjected to both univariate and multivariate ROC exploratory analysis. In Figure 2 among the nine metabolites related to MN-IgAN urine metabolome, univariate ROC analysis and their AUC values ranged from 0.8 to 0.77, suggesting that might be considered as moderate predictive biomarkers/metabolites. Among them, only six showed the highest discriminatory performance (Figure 2c) with the following area under the ROC curve (AUC) values: Indoxyl sulfate (7.25) value up to 0.846; Hippurate with AUC value at 0.803 (7.65); tartrate (4.35) and *N-*phenylacetylphenylalanine (7.19) with the lowest AUC value at 0.791 and 0.786, respectively (Figure 2d). Furthermore, the essential role in class discrimination is shown by the variables at 8.31 ppm and 7.27 ppm, which belongs to ^1^H NMR signals of inosine, imidazole and possibly exogenous metabolites (peptide’s NH (d) indicative ^1^H NMR signals) (Figure 2d). Biomarker evaluation procedure indicated that both variables present relatively high AUC values 0.821 and 0.801, respectively. Moreover, evaluation of the prediction of the performed multivariate model, PLS-DA, and a multivariate ROC explanatory analysis were performed (Figure 2a). To achieve this, the importance of metabolic features (VIP scores’ values) was assessed. The result is a panel of ROC curves of PLS-DA models’ performance, which utilize a combination of more than one discriminant metabolite/or ^1^H NMR spectral feature, selected via PLS-DA classification method (Figure 2b). The ROC curve is calculated using 25 features/metabolites on the PLS-DA model validation, presenting an AUC value of 0.807 (Figure 2a).

Multivariate analysis suggests five metabolites in predicting and distinguishing the IgAN from MN through urine metabolome. Indoxyl sulfate, hippurate, imidazole, *N*-phenylacetylphenylalanine and tartrate are found significantly higher in IgAN patients’ urine. Indoxyl sulfate is a uremic toxin that belongs to the protein binding low molecular weight compounds, which gradually increases in serum with the severity of CKD and thus has been positively correlated with the progression of CKD [26,27]. Hippurate is one of the most common organic anions, taking up almost 25% of the organic anion transport system (OATS) capacity in a healthy kidney. The endogenous clearance of hippurate in uremic rats is an indicator of changes in renal secretion associated with reductions in OATs protein expression in chronic renal failure, according to in vivo studies [28]. However, this is the first time that tartrate is reported as a significant metabolite discriminating the two glomerulonephritis subtypes. Most research studies suggest that urinary levels of tartrate are attributed to dietary sources and more specifically are a useful indicator of grape product consumption. Hence, this result might be a characteristic of the selected population. A few reports are also referring to *N*-phenylacetylphenylalanine as a urinary metabolite. However, since it can be assumed to be a derivative of phenylalanine, this suggests the involvement of phenylalanine’s metabolism, which is implicated in CKD biochemistry [29]. Park et al. propose urinary glycine levels as a potential biomarker for discriminating IgAN patients from patients with MN or other types of glomerulonephritis, however, in our study glycine did not represent any statistical significance in classification [12].

### 2.2. Chronic Kidney Disease Diagnosis as a Result of Other Systemic Diseases and its Relation with Membranous and IgA Nephritis

**Figure 3:**
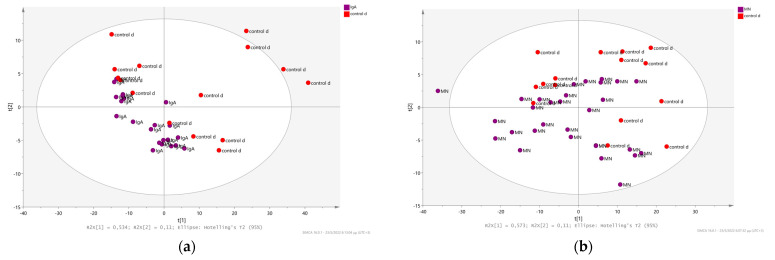
Urinary metabolic fingerprint comparison of Glomerulonephritis subtypes and control disease patients. Schemes follow another format. If there are multiple panels, they should be listed as: (**a**) PLS-DA 2D scores plot of ^1^H CPMG data matrix showing the separation between the urine fingerprint of IgAN (purple spheres) and control disease (orange spheres); (**b**) PLS-DA 2D scores plot of ^1^H CPMG data matrix showing the separation between the urine fingerprint of MN (purple spheres) and control disease (orange spheres).

Patients are often diagnosed with glomerulonephritis, however this phenotype is a result of other systemic diseases like lupus erythematosus, hematopoietic system’s diseases, diabetes mellitus and arterial hypertension. Such a disease onset leads to the presence of morphologically lesions in the glomeruli and it is clinically placed under the same CKD cause. Thus, it is interesting to examine if the urine metabolome from patients with systemic diseases (control disease group) might have the same pattern and/ or present variance-by-cause. To investigate the extent and the potential of urinary metabolic fingerprint in discriminating patients diagnosed with IgAN and MN from patients with glomerulonephritis originating from other systemic diseases, their ^1^H NMR data were subjected into comparative multivariate analysis. Fifteen patients (*n* = 15) diagnosed with glomerulonephritis from other systemic disease, annotated as control disease, were selected and compared with patients diagnosed with (a) IgAN and (b) idiopathic MN. Regarding the discriminative characteristics of the IgAN urine metabolome, NMR spectra were analyzed and compared to spectra from control disease. More specifically, normalized and transformed ^1^H CPMG NMR spectral data were used to construct a PLS-DA model. IgAN urine metabolome exhibit a unique metabolic pattern, leading the distribution of the class to a very narrow space with respect to the metabolome of the control disease (Figure 3a). The PLS-DA model presents good performance values R2X(cum): 0.705, R2Y(cum): 0.866 and Q2(cum): 0.299, and then was validated resulting in R2Y intercept: 0.802 and Q2Y intercept: 0.0858. VIP scores and PLS-DA coefficients show a reduction in levels of trigonelline (9.09) and 1-methylnicotinamide (8.93), while an increase at levels of xanthosine (5.81), tartrate (4.29), overlapping area (4.21), threonine and uridine ^1^H signals (4.25), Isobutyrate (1.09), 2-Hydroxybutyrate (1.59), creatinine (4.01) and UDP-glucose (4.39), uracil (5.79), cis-aconitate (5.71…5.67) and citrate (2.67) in urine metabolome of IgAN patients compared to control disease (Supplementary Figures S3a and S4a).

Comparison of the MN urine metabolome with the metabolic profile of control disease, revealed lower discrimination power and a fused distribution of the ^1^H NMR urine spectra on the multivariate variable space (Figure 3b). PLS-DA model results to moderate discrimination and classification values R2X(cum): 0.763, R2Y(cum) 0.759 and Q2: 0.347. To validate the model’s performance permutation test performed and resulted in R2Y intercept: 0.733 and Q2Y intercept: 0.0607. PLS-DA VIP scores and coefficients’ plot show that the main differences focus on lower levels of indoxyl sulfate (7.21), 3,4-Dihydroxymandelate (6.81), L-glutamate (2.29), exogenous metabolite lisinopril (2.33), ATP (8.27), creatinine (3.09), *N*-phenylacetylglycine (7.33), histidine (7.07), imidazole (7.45), 3,4-Dihydroxymandelate (6.81) and increase levels of *N*, *N*-dimethylglycine (2.93) in MN patients with respect to patients with control disease (Supplementary Figures S3b and S4b).

### 2.3. Comparison of the Metabolic Fingerprint of Hypertensive Nephrosclerosis (HN) and Diabetic Nephropathy (DN)

**Figure 4:**
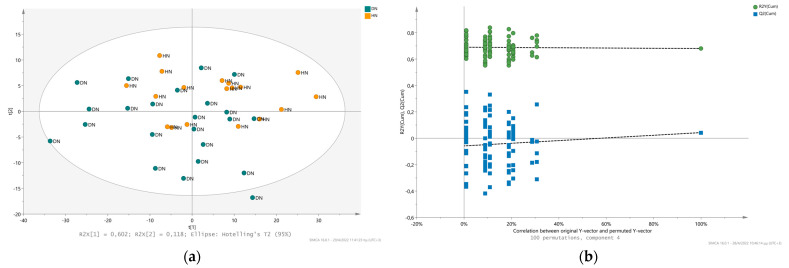
Urinary metabolic fingerprint Hypertensive Nephrosclerosis (HN) and Diabetic Nephropathy (DN): (**a**) PLS-DA 2D scores plot of ^1^H 1D NOESY data matrix showing the clustering between HN (orange spheres) and DN (blue spheres) urine fingerprint; (**b**) Permutation plot of the PLS-DA model validating the statistical significance of the model as the permuted R^2^ (green cycles) and Q^2^ (blue squares) are lower than the original values of R^2^ and Q^2^.

To date, metabolomic investigation of Diabetic Nephropathy and Hypertensive Nephrosclerosis via NMR is largely unexplored and their pathological comparison remains unclear. Throughout this study, 41 urine metabolic profiles have been analyzed, 18 of which were collected and recorded from patients with HN and 23 from patients with DN. Initial investigation of the urinary metabolic pattern performed via PCA analysis resulting in relatively overlapped distribution. The increased heterogeneity of ^1^H NMR spectra is imprinted to the PCA space, leading to low percentage (49.4% at the 3rd component) of the model’s explained cumulative variance (PCs). Furthermore, the PLS-DA model revealed low discrimination efficacy in classification of the two urine metabolic profiles with R2X(cum): 0.8, R2Y(cum): 0.68 and Q2(cum): 0.0429 (Figure 4a). To validate and assess the quality of the PLD-DA model, the permutation test performed and resulted in R2Y intercept: 0.69 and Q2Y intercept: −0.058, which both are not much lower than their original values (Figure 4b). These values indicate an over-fitted model, which cannot provide a reliable classification for the discrimination of diabetic nephropathy and hypertensive nephrosclerosis urine NMR fingerprint. To further evaluate the metabolic data, univariate statistical analysis of 17 common metabolites (which did not present any overlapping features in ^1^H NMR spectra) was performed. The metabolites’ concentration levels were compared for statistical significance, but none of them presented any statistical significance. Fold change analysis indicated the relative changes between DN and HN individuals, which are highlighted in Table 2. Only myo-inositol, citrate and 3-methyl-2-oxovalerate presented lower levels in the metabolome of HN patients, while sarcosine, 1-methylnicotinamide, hippurate, creatinine, trigonelline, isoleucine, 1-3-dihydroxyacetone, NAD^+^/NADP^+^, τ-methylhistidine, allantoin, acetone, alanine, lactate, and valine exhibited higher levels in HN with respect to DN patients (Table 2). Indeed myo-inositol has been reported as a metabolite associated with the diabetic nephropathy in animal models and has also been annotated as a uremic retention solute associated with progression to end-stage renal disease in diabetes type 2 patients [30,31]. Higher levels of citrate in DN urine metabolome support its importance and association with the pathology. Citrate is one of the most essential metabolites influencing the overall metabolic acidosis representing the endogenous acid production, which is strongly associated with the progression of the disease especially in diabetic nephropathy patients [32]. Additionally elevated 3-methyl-2-oxovalerate in urine suggests the presence of abnormal BCAAs breakdown, which has been reported by and is in accordance with its prevalence in diabetic patients since their metabolism moderately resembles the obese and cardiovascular metabolism, which is characterized by elevated serum BCAAs and low BCAAs catabolism [33,34,35].

## 3. Materials and Methods

### 3.1. Population Samples

Urine as a biological fluid is considered as the optimal biofluid for the studies of urological diseases and a variety of infections, dysfunctions and abnormalities including cancer. Since CKD is a pathophysiology, which reflects the progress of kidney dysfunction and a set of kidney morbidities, urine is selected as the biofluid of interest due to the non-invasive manner of collection, ease of sampling, chemical composition stability and particularly its rich composition of metabolites. In the present study, 108 ^1^H NMR spectra of urine were analyzed. ^1^H NMR spectra reflect the metabolic profile of urine samples from 30 Membranous Nephropathy, 22 with Immunoglobulin A Nephropathy, 23 Diabetic Nephropathy, 18 with Hypertensive Nephrosclerosis and 15 Control Glomerulonephritis disease patients. Individuals gave informed consent for the research, which was approved by the Ethical Committee of University Hospital of Patras. Every patient underwent general clinical testing and none of them presented any other disorder at the time of collection or chronic morbidity. The samples were collected at the General University Hospital of Patras. Gender was not a choice. Additional information (cause of disease, stage, comorbidity, eGFR value, sex and medication) was also recorded for every individual in the study (Table 3).

### 3.2. Sample Collection

The second morning urine samples were collected (3 to 5 mL) and centrifuged at 3000× *g* for 5 min at 4 °C. The supernatant was collected from each sample (divided into three portions of 1 mL in 2 mL cryovials) and stored at −80 °C until NMR analysis, according to standard operational procedures (SOPs) [37]. NMR preparation. Each sample (1 mL) was thawed at room temperature and was centrifuged at 14.000× *g* for 10 min at 4 °C. For each sample, 540 µL of urine the supernatant was mixed with 60 µL of potassium phosphate buffer (1.5 M KH_2_PO_3_, 100% *v*/*v* D_2_O, 0.05 mM 4,4-dimethyl-4-silapentane-1-sulfonic acid (DSS), 4%NaN_3_; pH 7.4) to a final total volume of 600 uL. After vicious vortex mixing, each sample mixture (600 µL) was transferred into a 5 mm NMR tube (Bruker BioSpin GmbH, Rheinstetten, Germany) for the analysis.

### 3.3. NMR Analysis 

The experiments were performed at the Department of Pharmacy of Patras University. Analysis of the samples was performed using a Bruker Avance III HD 700 MHz NMR spectrometer equipped with a cryogenically cooled TCI probe. The samples remained in the probe for at least 3 min, for temperature stabilization at 300K, before each measurement. For each urine sample, one-dimensional ^1^H NMR NOESY presat experiment with “presaturation” routine for water suppression (with 64 number of scans, 2 sec relaxation delay and 100ms mixing time) and one-dimensional ^1^H NMR Carr–Purcell–Meiboom–Gill (CPMG) with water presaturation (with 32 number of scans, 4 sec relaxation delay, total spin echo delay: 0.3 ms and loop for T2 filter 126) and a two-dimensional (2D) experiment ^1^H *J*-resolved were performed (using 4 number of scans per 128 increments for F1 (spin–spin coupling constant axis) and 12.3 K data points for F2 (chemical shift axis) [38,39].

### 3.4. NMR Data Processing 

Processing of the NMR spectra was performed using TopSpin 3.2 software (Bruker BioSpin GmbH). The ^1^H 1D CPMG spectrum was recorded for each urine sample to “filtrate-out” all the broad resonances that derive from proteic components (proteinuria, albuminuria). 1D ^1^H CPMG spectra were processed to remove phase and baseline distortions. Furthermore, all spectra were calibrated on DSS reference peak at 0.00 ppm, for multivariate and univariate statistical analysis. Segmentation of ^1^H 1D CPMG spectra was followed, constructing a matrix with buckets of 0.02 ppm spectral width. The urea and water protons resonance between 6.07–5.59 and δ_H_ 4.39–5.17 ppm, respectively, have been removed from each spectrum prior to statistical analysis. Normalized data implemented for univariate statistical analysis. NMR spectra segmentation was performed using AMIX software version 3.9.12 (Bruker BioSpin GmbH, Rheinstetten, Germany), while NMR Suite Chenomx software (Profiler 8.1 Chenomx Inc., Edmonton, AB, Canada), data from the Human Metabolome DataBase (HMDB) and literature were used to identify the metabolites [40].

### 3.5. Statistical Analysis

In multivariate statistical analysis, Probabilistic Quotient Normalization (PQN) and autoscaling were selected as methods for the best statistical preprocessing [41]. To obtain a first picture of the data, the statistical method of principal components analysis (PCA) was used. The partial least squares—discriminant analysis (PLS-DA) was chosen as the main supervised method for reducing the number of variables, aiming classification performance of the examined groups. OPLS-DA model for the IgAN and MN patients were also performed, yet RMSEE and RMSEP values indicated an over-fitted model. Multivariate analysis was performed using SIMCA 16.0.1 (Umetrics, Umeå, Sweden) and the online free available analytical platform MetaboAnalyst [42]. For statistical correlation purposes of the statistical significant variables, a mono-dimensional (1D) statistical total correlation spectroscopy analysis (STOCSY) was performed using the muma R package [43]. Univariate statistical approach was followed for the metabolic relative concentration calculation. Univariate analysis was applied running an in-house R script build for ^1^H signal area calculation. Wilcoxon rank sum test and the False Discovery Rate (FDR) correction was performed to determine the statistically significant metabolites (adjusted *p*-value < 0.05). For each, comparison fold changes of selected metabolites were performed as well. The R programming language (3.2.2) and the R studio software was used as computing tools. Receiver Operating Characteristic (ROC) curves were constructed using MetaboAnalyst for the PLS-DA multivariate model. The classification performance (sensitivity and specificity) of the biomarkers set in the identification on each glomerulonephritis etiology were assessed through the AUC.

## 4. Conclusions

To explore the potential of NMR metabolomics in differential diagnosis, we studied three of the most common etiologies of CKD using urine metabolic fingerprinting through NMR. Analysis of ^1^H 1D NMR spectra of urine samples from patients with IgAN, idiopathic MN, HN and DN has provided an insight into the similarities and differences of their urine metabolomes. The CKD subtypes with a discriminative potential appear to be the urine metabolomes of idiopathic MN and IgAN patients. Metabolomic data of DN and HN patients show that both pathologies present heterogeneous urine metabolism. This characteristic might be a result of the limited patient group along with their differences in medications and the presence of underlying co-morbidities. Hence, we suggest the urge of a larger cohort and the correlation analysis of metabolomic data with clinical parameters, specific biomarkers, and other histological features.

This is the first study that provides qualitative and quantitative differences of characteristic metabolites between and within the selected CKD subtypes, incorporate biochemical information regarding the initial cause of CKD. The reliability of the metabolomics results may be further enhanced by other analytical techniques and the use of standard references, and this will facilitate validation on large, independent cohorts and populations from other geographic locations.

## Figures and Tables

**Table 1:** ^1^H NMR Chemical Shifts and multiplicities of identified urine metabolites in CKD patients. The chemical shifts for each metabolite correspond to the ^1^H peaks used for their statistical correspondence with those that contribute to the urinary metabolic pattern.

No	Metabolites	^1^H NMR Chemical Shifts *
1.	1-methylhistidine	7.91 (m)
2.	Trigonelline	9.25...9.11 (s), 8.84…8.80 (m), 8.08…8.06 (m), 4.43…4.42 (s)
3.	1-methylnicotinamide	9.26 (s), 8.96…8.93 (d), 8.89…8.87(d), 4.46 (s)
4.	NADH/NADPH	8.46 (s), 8.17 (s), 6.28 (d), 5.94 (d)/ 2.79, 2.77 (s)
5.	NAD^+^/NADP^+^	9.35/8.37 (s), 6.04 (d), 6.06 (d)/ 8.43 (s), 6.06 (d), 6.09 (d)/
6.	ATP, AMP	8.55 (s), 8.26 (s)
7.	Hippurate	7.83…7.81 (d), 7.64….7.60 (m), 7.55…7.52 (m)
8.	Indoxyl sulfate	7.35 (s), 7.28…7.25 (m), 7.20… 7.16(m)
9.	Histidine	7.07 (m)
10.	3-Hydroxymandelate	7.31…7.27 (m/t), 6.97 (m), 6.90 (m), 6.85…6.82 (m)
11.	Anserine (-NH)	8.1 (s), 7.1 (s), 4.5 (m)
12.	Glycolate	3.94…3.97 (s)
13.	2-Hydroxyisobutyrate	1.3 (s)
14.	Tartrate	4.3 (s)
15.	creatinine	4.04 (s), 3.03 (s)
16.	Mannitol	3.87…3.84 (dd), 3.80…3.78 (m), 3.77…3.73 (m), 3.69…3.66 (m)
17.	Myo-inositol	4.06 (m), 3.68 (br), 3.55–3.54 (dd)
18.	*sn*-glycerol-	3.65 (m)
19.	TMAO	3.25 (s)
20.	Sarcosine	2.76 (s) broad- 3.60 (s) overlapped
21.	*N*-phenylacetylglycine	7.43…7.40 (m), 7.36…7.33 (m)
22.	*N*-phenylacetylphenylalanine	7.75(d), 7.31…7.26 (m), 7.18…7.15 (m), 7.10…7.08 (m)
23.	Gentisate	7.29…7.27 (overlapped) 6.98…6.96 (dd), 6.86…6.84 (d)
24.	3, 4-Dihydroxymandelate	6.91…6.87 (m), 6.84…6.82 (dd)
25.	Salicylate	7.85 (dd), 7.47 (m), 6.97…6.95 (m)
26.	Trans-Aconitate	6.59–6.57 (s)
27.	Xanthosine	7.9 (s), 5.81 (d)
28.	uracil	7.5 (d), 5.77 (d)
29.	urea	5.75 (s, br)
30.	cis-Aconitate	5.71…5.65 (m)
31.	Allantoin	5.38…5.36 (s), br
32.	1,3- Dihydroxyacetone	4.42…4.41
33.	UDP-glucose/	4.39 (m)
34.	Glucose	3.25…3.24
35.	Taurine	3.44…3.41 (t), 3.27…3.24 (t)
36.	Citrate	2.70...2.67 (d), 2.56…2.52 (d)
37.	*N*, *N*-Dimethylglycine	2.93…2.91 (s)
38.	*N*-methylhydantoin	4.1 (s), 2.9 (s)
39.	2-hydroxybutyrate	1.70...1.63 (m), 1.63…1.57 (m)
40.	Betaine	3.9 (s), 3.3 (s)
41.	Lysine	3.01...2.9 (t), 1.92...1.87 (m)
42.	Gamma-aminobutyrate	3.00…2.97 (t), 1.94…1.90 (m)
43.	Proline	1.94…1.92 (m)
44.	Isoleucine	0.99…0.97 (d)
45.	Leucine	0.96…0.94 (d), 0.95…0.93 (d)
46.	Valine	1.04…1.02 (d), 0.98…0.97 (d)
47.	3-aminoisobutyrate	1.19…1.17 (d)
48.	3-methyl-2-oxovalerate	1.07…1.05 (d), 0.89…0.86 (t)
49.	Isobutyrate	1.10…1.08 (d)
50.	Alanine	1.48…1.46 (d)
51.	Succinate	2.39 (s)
52.	Glucuronate	5.2 (d), 4.7 (d)
53.	Erythritol	3.8 (d), 3.7 (d)
54.	Lactose	5.22 (d)
55.	Uridine	7.9 (m), 5.9 (d), 4.39 (m), 4.25 (m)
56.	L-Glutamate	2.37…2.30 (m)
57.	Sialic acid	2.33 (dd)
58.	Lisinopril (zestril)	7.40 (t), 7.32 (m), 2.33 (m)
59.	Aspartate	2.83 (dd)
60.	Pyroglutamate	4.19…4.15 (m)

* ^1^H NMR signals multiplicities: (s) singlet; (d) doublet; (t) triplet; (dd) doublet of doublets; (m) multiplet; (br) broad.

**Table 2:** Univariate analysis of common urine metabolic features in DN and HN individuals.

Metabolite	Raw *p*-Value	Log2 (FC) ^1^	Fold Change in DN/HN
Sarcosine	0.095	−1.158	0.44801 ▲^2^ HN
1-Methylnicotinamide	0.178	−0.976	0.50828 ▲ HN
Hippurate	0.290	−0.906	0.52994 ▲ HN
Myo-inositol	0.691	0.856	1.8102 ▼ HN
Creatinine	0.056	−0.720	0.60693 ▲ HN
Trigonelline	0.49	−0.714	0.60954 ▲ HN
Isoleucine	0.152	−0.720	0.60696 ▲ HN
1,3- Dihydroxyacetone	0.427	−0.717	0.60828 ▲ HN
NAD+/NADP+	0.866	−0.350	0.78433 ▲ HN
τ-Μethylhistidine	0.056	−0.749	0.59479 ▲ HN
Allantoin	0.119	−0.355	0.7817 ▲ HN
Citrate	0.664	0.156	1.1144 ▼ HN
Acetone	0.071	−0.353	0.78286 ▲ HN
Alanine	0.630	−0.205	0.86759 ▲ HN
Lactate	0.687	−0.072	0.95136 ▲ HN
3-methyl-2-oxovalerate	0.948	0.093	1.0665 ▼ HN
Valine	0.524	−0.698	0.61621 ▲ HN

^1^ FC with a positive value indicates a relatively higher concentration present in DN patients, and a negative value shows a relatively higher concentration in HN patients. ^2^ Symbols ▲ and ▼ indicate the elevated levels and the decreased levels for each metabolite in HN group.

**Table 3:** Characteristics of study participants.

Cause	Age	Sex (M/F)	Rate Decline eGFR	Urine Creatinine
Membranous Nephropathy	55.83	25/5	5.19 ± 61.94	78.63 ± 39.49
Immunoglobulin A Nephropathy	43.46	16/6	−3.94 ± 32.78	86.34 ± 49.25
Diabetic Nephropathy	68 ± 8.1	22/1	−0.64 ± 5.38	55.98 ± 44.57
Hypertensive Nephrosclerosis	59.9 ± 1.38	13/5	−3.92 ± 11.5	61.14 ± 48.21
Control Diseased Glomerulonephritis	57.13 ± 14.46	8/7	−6.17 ± 20.28	71.33 ± 48.88

Age, gender, rate decline eGFR and urine creatinine were determined to be the most important metadata. Estimated GFR was calculated using serum concentration of creatinine measured at baseline using the MDRD GFR Equation [36]. For eGFR and urine Creatinine, the mean and standard deviation are shown with the range.

## Data Availability

Data and R script are available from the corresponding authors upon reasonable request. The data are not publicly available due to privacy.

## Supplementary Materials

The following supporting information can be downloaded at: https://www.mdpi.com/article/10.3390/metabo12060490/s1, Figure S1: Membranous Nephropathy (MN) 1H NMR urinary metabolic profile. Unsupervised visualization of cluster tendency: (a) PCA 2D scores plot of MN applied on the data set of 30 1H CPMG NMR urine spectra; spheres are colored according to the eGFR value via heatmap visualization; (b) PCA-Hierarchical Clustering on the same MN metabolic NMR profiles; Figure S2: IgA Nephropathy (IgAN) 1H NMR urinary metabolic profile. Unsupervised visualization of cluster tendency: (a) PCA 2D scores plot of IgAN applied on the data set of 22 1H CPMG NMR urine spectra; spheres are colored according to the eGFR value via heatmap visualization; (b) PCA-Hierarchical Clustering of the IgAN metabolic NMR profiles; Figure S3. Statistically significant features of the glomerulonephritis subtypes comparison with the control disease patients. The first 10 VIP scores of the first PLS-DA Latent Variable indicating the variables of importance between (a) IgAN and (b) MN and control disease patients’ metabolic profile, respectively; Figure S4. PLS-DA coefficients’ plot for the classification, which reveals the relative intensities between the two examined groups: (a) IgAN and control disease and; (b) MN and control disease patients. The color-coded correlation coefficients indicate the significance of the metabolites in terms of their contribution to the separation between the two groups.
