# Supplementary material for: NMR-Based Metabolomics in Differential Diagnosis of Chronic Kidney Disease (CKD) Subtypes
Source: Metabolites. 2022 May 28;12(6):490. doi: 10.3390/metabo12060490 (PMC9230636; doi:10.3390/metabo12060490)
Supplement: Supplementary file 1 [file metabolites-12-00490-s001.zip › metabolites-1728187-supplementary.pdf]

NMR-based metabolomics in differential diagnosis of chronic kidney disease (CKD) subtypes

Supplementary Material

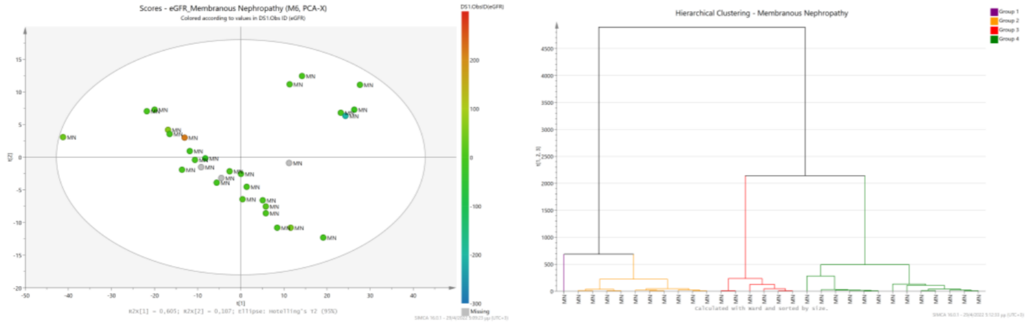

(**a**) (**b**)

Figure S1. Membranous Nephropathy (MN) ^{1}H NMR urinary metabolic profile. Unsupervised visualization of cluster tendency. (a) PCA 2D scores plot of MN applied on the data set of 30 ^{1}H CPMG NMR urine spectra; spheres are colored according to the eGFR value via heatmap visualization. (b) PCA-Hierarchical Clustering on the same MN metabolic NMR profiles.

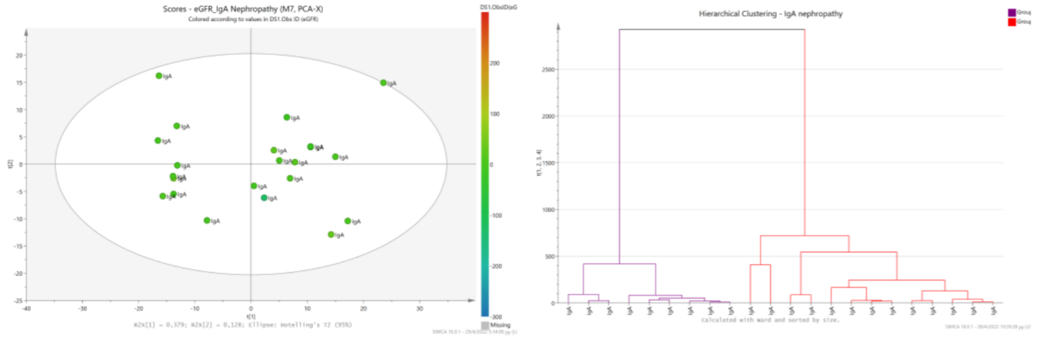

(**a**) (**b**)

Figure S2. IgA Nephropathy (IgAN) ^{1}H NMR urinary metabolic profile. Unsupervised visualization of cluster tendency. (a) PCA 2D scores plot of IgAN applied on the data set of 22 ^{1}H CPMG NMR urine spectra; spheres are coloured according to the eGFR value via heatmap visualization. (b) PCA-Hierarchical Clustering of the IgAN metabolic NMR profiles.

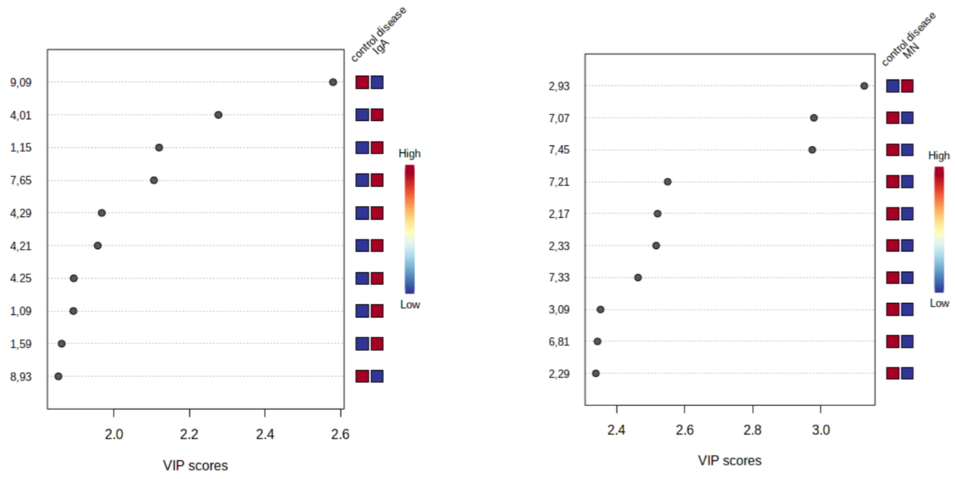

(**a**) (**b**)

Figure S3. Statistically significant features of the glomerulonephritis subtypes comparison with the control disease patients. The first 10 VIP scores of the first PLS-DA Latent Variable indicating the variables of importance between (a) IgAN and (b) MN and control disease patients' metabolic profile respectively.

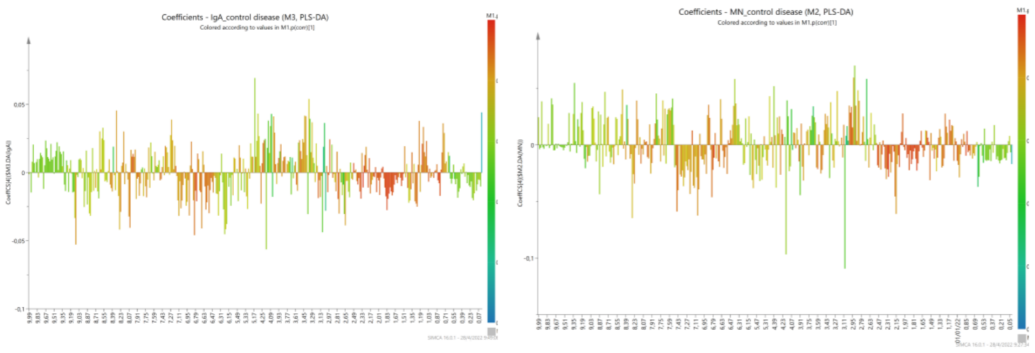

(**a**) (**b**)

Figure S4. PLS-DA coefficients' plot for the classification which reveals the relative intensities between the two examined groups. (a) IgAN and control disease and (b) MN and control disease patients. The color-coded correlation coefficients indicate the significance of the metabolites in terms of their contribution to the separation between the two groups.
